# Assessment of a storage system to deliver uninterrupted therapeutic oxygen during power outages in resource-limited settings

**DOI:** 10.1371/journal.pone.0211027

**Published:** 2019-02-06

**Authors:** Ryan Calderon, Melissa C. Morgan, Mark Kuiper, Harriet Nambuya, Nicholas Wangwe, Akos Somoskovi, Daniel Lieberman

**Affiliations:** 1 Intellectual Ventures Laboratory, Bellevue, Washington, United States of America; 2 Department of Pediatrics, University of California San Francisco, San Francisco, California, United States of America; 3 Institute for Global Health Sciences, University of California San Francisco, San Francisco, California, United States of America; 4 Maternal, Adolescent, Reproductive, and Child Health Centre, London School of Hygiene and Tropical Medicine, London, United Kingdom; 5 Department of Pediatrics, Jinja Regional Referral Hospital, Jinja, Uganda; 6 Intellectual Ventures Global Good Fund, Bellevue, Washington, United States of America; University of Science and Technology Beijing, CHINA

## Abstract

Access to therapeutic oxygen remains a challenge in the effort to reduce pneumonia mortality among children in low- and middle-income countries. The use of oxygen concentrators is common, but their effectiveness in delivering uninterrupted oxygen is gated by reliability of the power grid. Often cylinders are employed to provide continuous coverage, but these can present other logistical challenges. In this study, we examined the use of a novel, low-pressure oxygen storage system to capture excess oxygen from a concentrator to be delivered to patients during an outage. A prototype was built and tested in a non-clinical trial in Jinja, Uganda. The trial was carried out at Jinja Regional Referral Hospital over a 75-day period. The flow rate of the unit was adjusted once per week between 0.5 and 5 liters per minute. Over the trial period, 1284 power failure episodes with a mean duration of 3.1 minutes (range 0.08 to 1720 minutes) were recorded. The low-pressure system was able to deliver oxygen over 56% of the 4,295 power outage minutes and cover over 99% of power outage events over the course of the study. These results demonstrate the technical feasibility of a method to extend oxygen availability and provide a basis for clinical trials.

## Introduction

Globally, lower respiratory tract infections, including pneumonia, are a leading cause of mortality in children, accounting for over 700,000 deaths in children under age 5 in 2015 [1]. An estimated 120 million cases of childhood pneumonia, 14 million of which are severe, occur each year [2–9]. Oxygen is an essential therapy to treat hypoxemia [8], but remains under-utilized in many low-resource settings due in part to cost and implementation challenges [10]. Many health facilities lack or are unable to maintain reliable access to oxygen and related supplies [11–13].

Compressed gas cylinders are a common standalone system that do not require electricity to supply oxygen. However, they do require a system linking the oxygen production plant to the health facility to enable refilling, the reliability of which may be challenging due to poor road conditions, transportation costs, and stock management issues in low-income settings [14–16]. Leaks have been observed at connection points, leading to significant wastage [17,18].

Oxygen concentrators are a common alternative to cylinders that, with reliable electricity, can produce oxygen onsite continuously [17], alleviating the transport and supply issues common with cylinders. Multiple studies have evaluated use of concentrators in low- and middle income countries (LMIC) and shown them to be cost-effective compared to cylinders [19–27]. Key considerations to successful implementation require trained staff, provisions for maintenance, and reliable power [12,17,25,28–30].

Lack of access to reliable electricity is a problem in many low-resource settings [31], limiting the utility of oxygen concentrators. In facilities that lack reliable electricity, options to extend the use of oxygen concentrators include batteries and solar power. Studies in The Gambia and Uganda have demonstrated successful use of solar power for this purpose, with a back-up source required only rarely during periods of rain [32,33]. The major disadvantage of solar power is high capital cost, which ranged between $13,000 and $18,000 in these two studies, with solar panels comprising the bulk of this cost [32,33]. Planned trials in Uganda, Papua New Guinea, and Nigeria will provide additional evidence about the feasibility and efficacy of solar-powered oxygen systems [34–36]. Costs for a battery-powered system are also high initially but can be designed to successfully bridge power gaps [37]]. One analysis estimated that a battery-powered system should pay for itself within 12 months [38]; however, this has not been tested. There have been several attempts to store oxygen on site at relatively low pressures (< 10 bar). One commercially available oxygen storage system (Oxygen Reservoir Filling System, Diamedica, UK) can be filled from a concentrator and, more recently, a low-pressure oxygen storage bladder prototype has been tested in Uganda [39].

In order to improve oxygen supply in health facilities, Global Good/Intellectual Ventures has developed a novel Low-Pressure Reservoir (LPR) oxygen storage system that integrates with a concentrator to provide uninterrupted oxygen supply during power outages. This technology provides an alternative to existing oxygen storage that has the potential to increase coverage with minimal cost or complexity. The objectives of this non-clinical evaluation were to evaluate performance measures of a prototype device both in the lab and in a hospital setting, including the extent to which the LPR system provides uninterrupted oxygen during a power outage, the time required to replenish the reservoir, and oxygen quality and flow rate from the system. The field trial was carried out at Jinja Regional Referral Hospital (JRRH) over a 75-day period. A study at JRRH in 2014 recorded 120 episodes of power failure (mean of 13 times per week) during continuous monitoring over a 64-day period with a median duration of 30 minutes [33].

## Materials and methods

### Description of device

**Fig 1:**
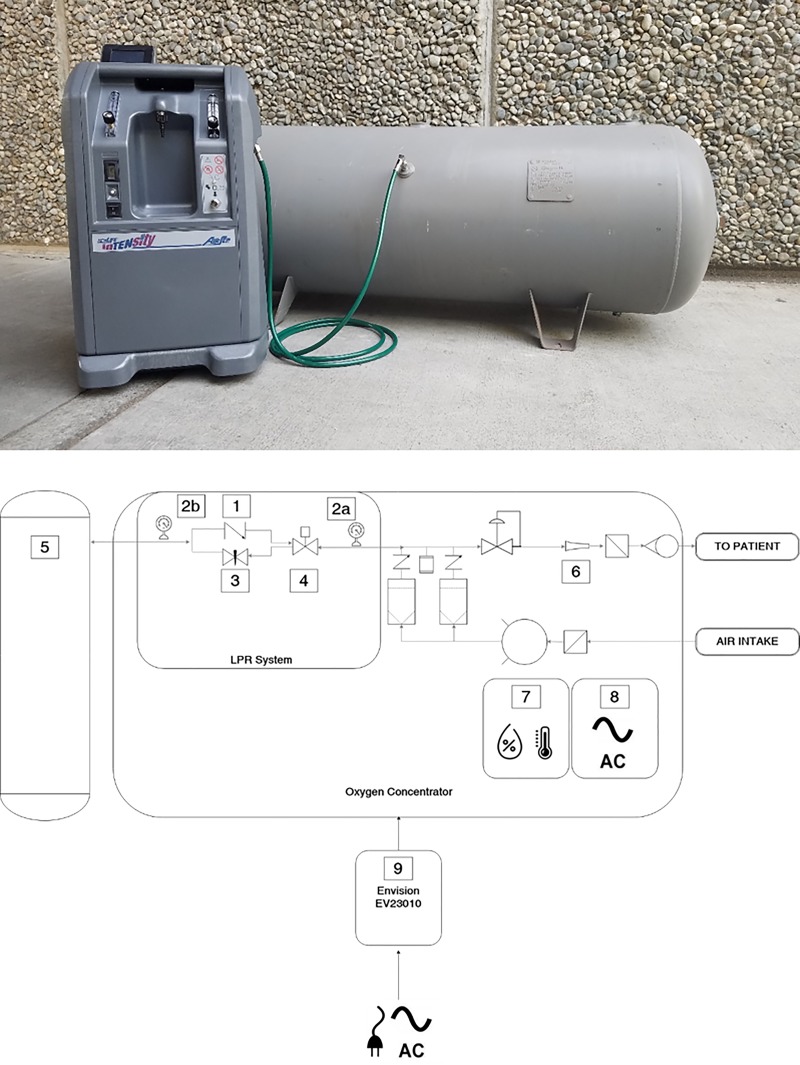
(A) Low-Pressure Reservoir system used in this study. (B) Low-Pressure Reservoir system is comprised of a reservoir and flow loop (1-check valve, 2-pressure sensor, 3-flow control orifice, 4-electrical solenoid, 5-reservoir, 6-flow meter, 7-temperature and humidity sensors, 8-power monitor, 9-surge protection) to store excess oxygen from the high pressure side of a concentrator. The sensors are added for monitoring purposes.

The Global Good/Intellectual Ventures’ LPR storage system consists of a 303-liter reservoir (Manchester Tank, Franklin, TN), tubing, and a flow metering system connected to a 10 liter per minute (LPM) oxygen concentrator (Airsep Intensity, Chart Industries, Ball Ground, GA) to provide oxygen continuously during power outages (Fig 1A). Using materials readily available in resource-limited settings, this storage device is designed to fill with excess oxygen that is generated by the concentrator, while maintaining adequate oxygen flow to up to two pediatric patients. Oxygen is delivered to the patient from the concentrator rotameters. The reservoir is connected to the high-pressure side of the concentrator via a flow metering system. The system consists of an orifice to limit flow into the reservoir and a check valve to enable unencumbered flow from the reservoir to the concentrator during a power outage (Fig 1B). The flow control orifice (7781K21, McMaster-Carr, Santa Fe Springs, CA), which was preset to divert at most 5 standard liters per minute (SLPM), meters oxygen to fill the reservoir. The maximum pressure in the reservoir is limited to 284 kPa (<1 LPM), the high side pressure generated by the compressor in the concentrator. The orifice is added to maintain the design pressure in the concentrators pressure swing absorption. A check valve (47245K25, McMaster-Carr, Santa Fe Springs, CA) allows oxygen to flow freely from the reservoir back into the concentrator. The pressure regulator inside the concentrator was set to an internal back pressure of 172.4 kPa.

When power is available, oxygen flows from the concentrator directly to the patient. If the reservoir is not full, oxygen will flow to reservoir and to the patient. During reservoir filling, patient flow is limited to 5 SLPM. When full, the unit can supply up to the concentrator’s maximum of 10 LPM. If there is a power outage, the stored oxygen will flow automatically through the flow metering system’s check valve into the concentrator and through the rotameters to patients. A shutoff valve was added to isolate the reservoir when the system is turned off.

A power protection device (Envision, EV23010) was used to protect the prototype. The unit was programmed to cut power to the concentrator if the supply line voltage deviated outside a 195–264 VAC safe range.

The LPR system was designed for oxygen use following appropriate design guidelines and standards [40,41]], and was cleaned to minimize particulate and non-volatile residues that can pose an ignition risk.

### Materials

Field data was collected using a compact data acquisition system and a suite of sensors mounted inside the oxygen concentrator. The sensors included: a pressure transducer (Honeywell, HSCMRR060PDSA3) measuring reservoir pressure, a flow meter (Honeywell, HAFUHT0010L4AXT) installed at the patient outlet of the concentrator upstream of the rotameter, temperature and relative humidity (Sensirion, SHT31-DIS-B) located on the custom PCB, and AC mains status of oxygen concentrator (MID400S). Oxygen concentration was measured at the concentrator patient outlet using and oxygen analyzer (O2Cap, Oxigraf, Sunnyvale, CA). All sensors were measured and logged to a local removable flash storage card (SD card) at an interval of 4 seconds. Once a minute, data would be serially communicated to a cellular modem (Particle Electron, 900/1800 MHz) that relayed the data to a cloud database. Data outages less than 5 minutes were filled with the last collected data point value. Data outages greater than 5 minutes were omitted from analysis.

### Study design

The study was carried out in two phases. First we evaluated the prototype baseline performance in the laboratory and then we operated at JRRH in Jinja, Uganda to evaluate operation in its target environment. In all tests, flow rate to the patient, reservoir pressure, oxygen concentration or oxygen alarm status, and power to the concentrator were measured. We then calculated power outage time, oxygen outage time, time to fill and drain the reservoir.

### Procedures

#### Baseline evaluation

Baseline operation of the prototype was evaluated in a laboratory setting (Intellectual Ventures Laboratory, Bellevue, WA) prior to field deployment at JRRH. The purpose of this test was to measure the basic operation of the prototype, namely that the reservoir is able to store oxygen and then provide continuous coverage during a power outage. In this test, the LPR system was evaluated over a 15-hour period, where power outages ranging from 15 to 90 minutes in duration were imposed. At the start of the test, the power was turned on and the rotameter flow rate was set to 5 LPM. The system was energized for a long enough time to allow the reservoir to replenish before introducing a simulated outage. When an outage occurred, the concentrator shut down and oxygen drained from the reservoir through the concentrator patient rotameter. When power was restored, the concentrator resumed operation and oxygen could flow through the rotameter and replenish the reservoir. Reservoir pressure, oxygen concentration and flow rate at the patient outlet were measured. From these data time to fill and drain the reservoir could be calculated.

#### Field evaluation

This study was conducted between April and July 2017 over a 75-day period at JRRH. The prototype was placed in the emergency room supply closet connected to an electrical outlet with the reservoir located outside in a secure cage. A 10-meter hose connected the reservoir to the concentrator. The purposes of the field evaluation were to measure the basic operation of the prototype in a hospital connected to an unreliable power grid and to evaluate the extent to which the system could supply oxygen during a power outage.

A power outage was defined to occur when the mains voltage deviated from nominal 230VAC RMS ± 15% (outside 195V to 264V range). This included both interruptions from the grid and periods when the generator was not used. An oxygen outage was defined when the reservoir pressure was less than 115.1 kPa and there was a power outage. The power outage coverage was defined as the number of minutes oxygen was delivered from the LPR system relative to the total power outage. If zero power outage minutes were recorded then coverage percent is null. A coverage outage resulted when an AC mains outage occurred and the reservoir pressure fell below the set regulator pressure, as the rotameter flow rate indication would have been out of calibration. This underestimates patient coverage slightly, as oxygen will continue to flow at ever decreasing flow rates until the reservoir completely drains.

Gaps in the data were periods that the prototype was offline or not recording data, and these are not included in the figures or analysis. There were a total of 15 calendar days with complete data gaps (May 7, 9, 17–18; June 5–11, 17–18, 23–24), as shown in S1 Fig. On days with partial data coverage, the power outage statistics were normalized to recorded minutes.

The patient flow rotameters were adjusted on a weekly basis throughout the study between 0.5–5 LPM to mimic different treatment scenarios. Weekly oxygen concentrator maintenance, as recommended by the manufacturer, included gross particle filter inspection and cleaning and monthly maintenance included replacing the local data storage card.

The tank was cleaned using a tank roller setup, industrial cleaner (Simple Green, Sunshine Makers, Huntington Beach, CA), and triangular deburring smooth ceramic media (4918A75, McMaster Carr, Santa Fe Springs, CA). Oxygen quality, including possible impurities generated from within the tank were measured before tank cleaning (May 2016), during setup after tank cleaning in Jinja, Uganda (June 2016), and during decommissioning at the end of the study period (July 2017). Collected samples were tested (TRI Air testing, Austin, TX) to compare to concentrator oxygen standards.

### Ethics

This study did not require ethical approval as it did not involve human or animal subjects. The prototype concentrator and reservoir tank were installed at JRRH prior to the start of the study, with the tank installed outside of the hospital walls in an access-controlled area.

## Results

### Baseline evaluation

**Fig 2:**
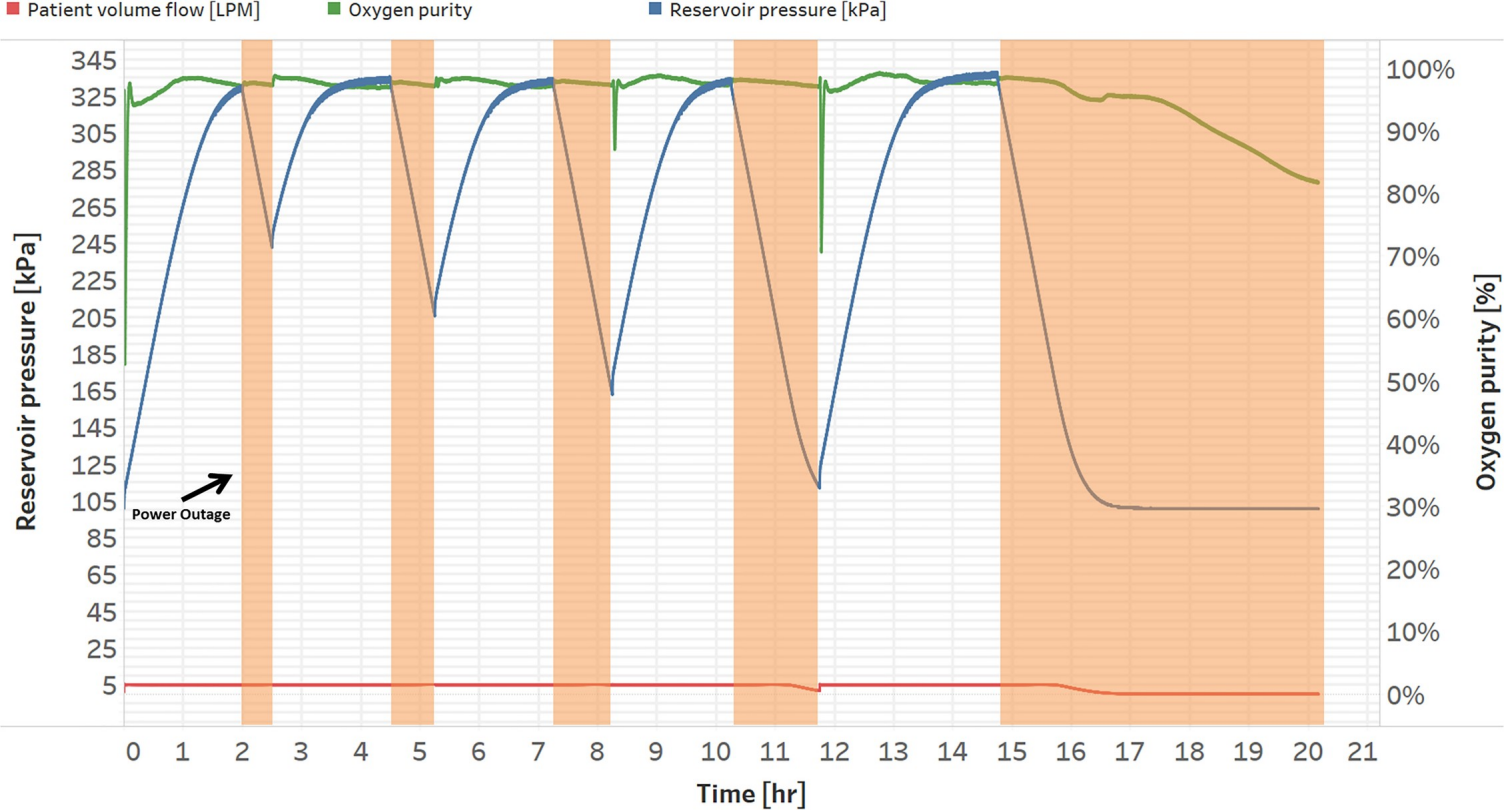
Laboratory test of the low-pressure reservoir system. Power outages were imposed on the system with intervals of 15 to 90 minutes in duration. The system flow rate was set at 5 liters per minute.

Basic operation of the device as reported by the oxygen flow rate, oxygen quality, and reservoir pressure is shown in Fig 2. The system maintained a steady flow of oxygen with no noticeable changes in quality or needed rotameter flow adjustment when a power outage occurred. When power was restored, the concentrator resumed flow to the patient and replenished the reservoir. During a power outage the reservoir pressure decreased as oxygen exited the rotameter. When the reservoir pressure dropped below the internal regulator back pressure (115.1 kPa), the flow rate to the patient was observed to decrease and eventually stop when the reservoir emptied. Oxygen quality was maintained above 90% for the majority of the evaluation. Two drops in oxygen quality were observed at 8.2 and 11.8 hours when power was restored to the prototype. The magnitude of the drop was observed to increase with a more depleted reservoir. We noted that oxygen quality from an off-shelf concentrator required several minutes to reach above 82% concentration during startup and the oxygen alarm was typically tripped during this period. Interestingly, with the LPR system, low quality oxygen during concentrator startup was not observed following the first two power outages of shorter duration. We suspect that the reservoir system maintained a sufficient back pressure to the pressure swing absorption (PSA) to produce higher quality oxygen [42]. Filling an empty 303 L reservoir required 190 minutes.

### Field evaluation

**Fig 3:**
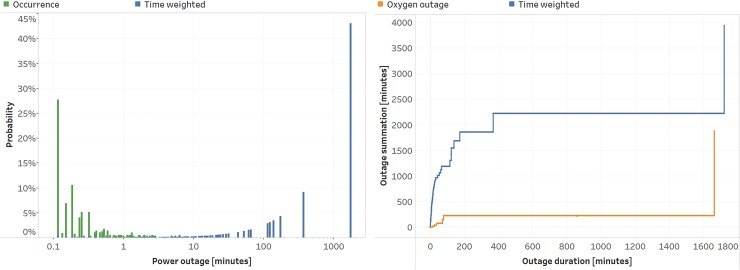
(A) Probability density of power outage events by occurrence and weighted by total outage time. (B) Cumulative power outage minutes (4295 min) and oxygen outage minutes (1884 min) remaining after the depletion of the Low-Pressure Reservoir are plotted as a function of individual outage duration events over the study period.

During the 75-day period of power monitoring at JRRH, a total of 1,284 power outage episodes (voltage outside 200-264VAC and lasting for > 4 seconds) with mean duration of 3.1 minutes (range 0.08 to 1720 minutes). Power outages accounted for 5.2% of time over the course of the study. Fig 3A shows the probability of a power outage weighted by occurrence and by outage time. The majority of power outage occurrences (98%) were short in duration, lasting less than 100 minutes, but accounted for only 13% of total power outage time. Infrequent, longer power outages, lasting greater than 400 minutes, accounted for more than 85% of total power outage time.

Fig 3B plots a summation of power outage and oxygen outage as a function of outage events. At each power outage event, the total number of minutes includes all the events of shorter duration (to the left) forming a staircase-like plot. Vertical line segments represent outage events. Horizontal line segments represent no outage event over the spanned duration. The total power outage and oxygen outage time over this study is the last point on the right end of each trace. Two observations are made comparing oxygen to power outages. First, the total oxygen outage time (1,884 minutes) was less than half the power outage time (4,295 minutes). Second, the majority of the reduction was observed for power outage durations less than 400 minutes, which represented over 90% of power outage events. The single event at approximately 1,700 minutes outage duration was only partly covered using the prototype. The power outage trace is a proxy for oxygen outages of an off-the-shelf stationary concentrator since it would not function during a power outage. Over the trial period, the LPR prototype experienced 11 oxygen outage events compared to 1,284 events that an off-the-shelf concentrator would have experienced in this study.

**Fig 4:**
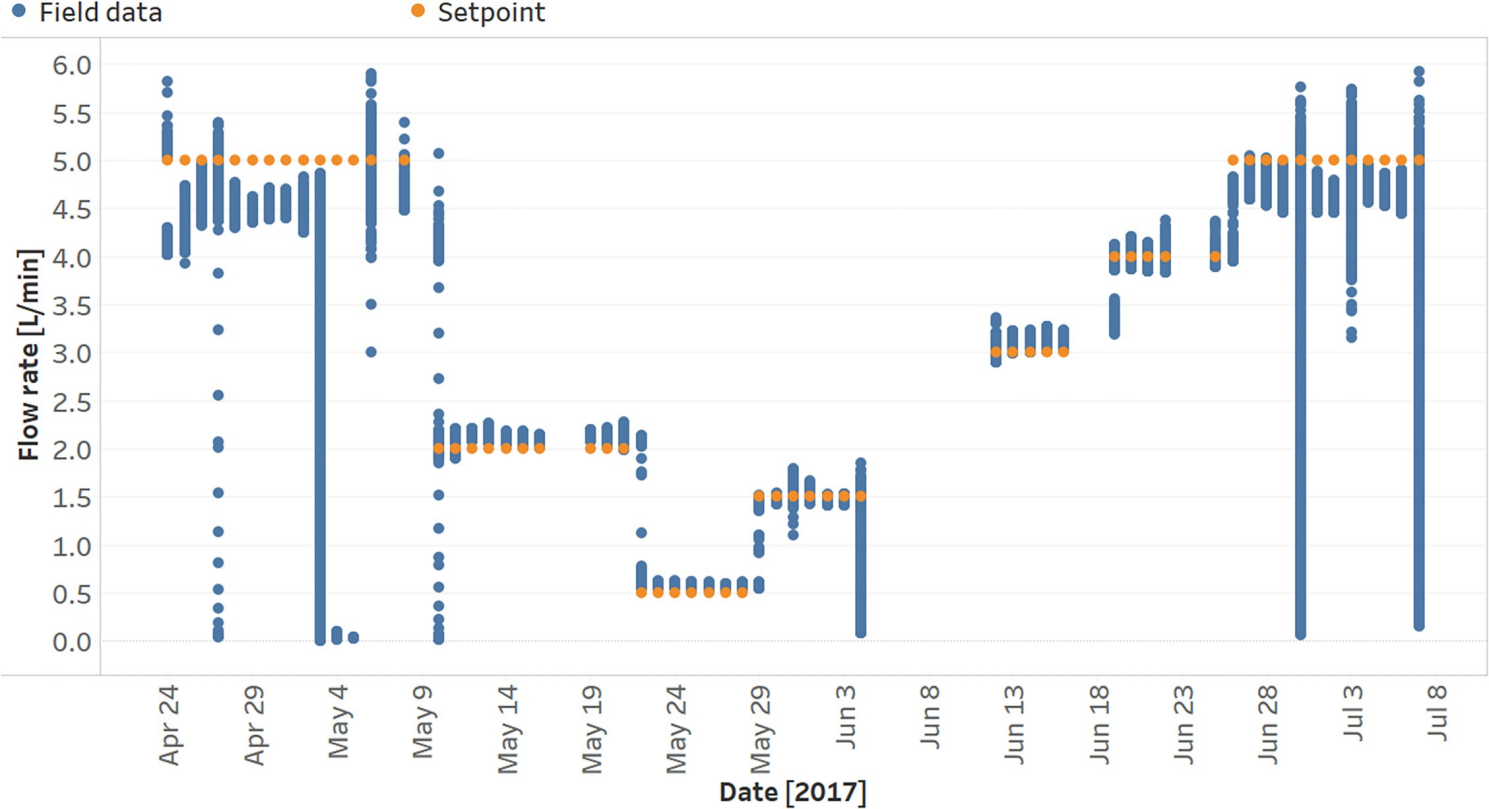
Actual and set point flow rates over the study period.

Flow rate measured at the patient rotameters are plotted, with set point flow rates overlaid for comparison, in Fig 4. In this figure, drops in flow rate correspond to low reservoir pressures after a prolonged power outage. The temperature (M = 37.2°C, SD = 3.9°C) and relative humidity (M = 39.3%, SD = 7.6%) inside the prototype were recorded over the study period.

**Fig 5:**
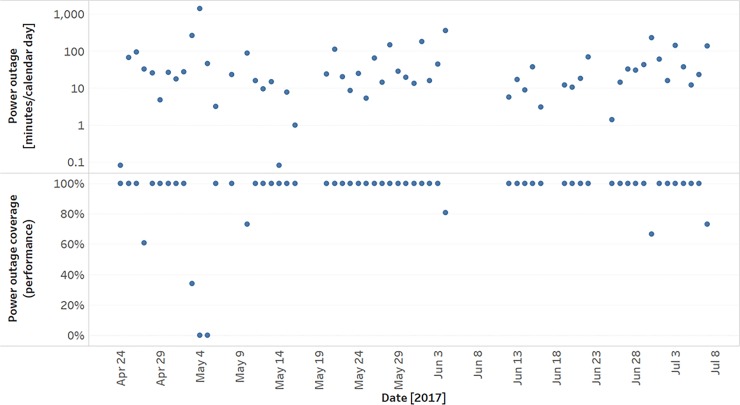
Daily power outage duration and associated low-pressure reservoir system performance. (A) Cumulative power outage minutes per calendar day. (B) Low-Pressure Reservoir system power outage coverage shown for each day.

Cumulative daily power outage durations over the course of the study are plotted in Fig 5A. Each daily value is a summation of the power outage events over a calendar day. The power outage coverage, the number of minutes oxygen was delivered from the LPR system relative to the total power outage minutes per day, is shown in Fig 5B. The LPR system provided 100% oxygen coverage for 51 calendar days during the study. On these days, the power outage duration averaged 32.9 minutes (range 1 to 184 minutes), with an average flow rate of 3 LPM (range 0.5 to 4.7 LPM). On 8 calendar days with oxygen outages, the power outage duration averaged 326.7 minutes (range 33.4 to 1440 minutes), with an average flow rate of 3.6 LPM (range 0 to 4.6 LPM).

Oxygen quality test results, shown in Table 1, meet all categories of gases for use in medical applications. The gas was sampled over a thirteen-month period between May 2016 and June 2017 during which the unit operated continuously, delivering over 413,750 L of oxygen. No increases in particulate, oil mist, or hydrocarbons were observed. These results support using a commercial, off-the-shelf, steel tank (cleaned) for therapeutic oxygen storage.

**Table 1:** Low-pressure reservoir gas quality testing and standards.

	Oxygen (volume %)	Carbon Monoxide (ppmv)	Carbon Dioxide (ppmv)	Oil Mist (mg/m3)	Particulate (mg/m3)	Nitrogen Dioxide (ppmv)	Sulfur Dioxide (ppmv)	Water (ppmv)	Water (Dew Point°F)	Nitric Oxide (ppmv)
**Baseline** **May 23, 2016**	94.2	<1	<1	<0.03	<0.03	<0.03	-	460	-19	-
**Setup** **June 21, 2016**	91.9	<1	<25	<0.02	<0.02	<0.1	<0.1	163	-35	<0.1
**Decommission** **July 15, 2017**	89.0	<1	34	<0.02	<0.02	<0.1	<0.1	460	-19	<0.1
**Ph. Eur. Oxygen 93**	90.0–96.0	< 5	300	0.1	N/A	<2	<1	<67	N/A	<2
**USP Oxygen 93 Percent**	90.0–96.0	10	300	N/A	N/A	N/A	N/A	N/A	N/A	N/A

## Discussion

This study evaluated the technical performance of the LPR prototype in the laboratory and field settings. The data demonstrated that the prototype supplied oxygen to cover over half of the recorded power outage minutes over the flow rates set between 1–5 LPM.

Oxygen storage systems typically tradeoff footprint at the expense of storage capacity. The 303 L reservoir used in this study can store 542 standard liters of oxygen (273 kPa max), enabling oxygen delivery for 108, 271, and 542 minutes (5, 2, 1 LPM flow rates, respectively). The prototype could accommodate a range of reservoir sizes and could be used with most 5–10 LPM stationary concentrators. A look at Figs 4 and 5 indicates, in general, fewer drops in flow rate were observed at lower set points, which is expected due to the longer coverage times available from the reservoir.

The LPR prototype architecture was selected to minimize additional components and leverage the available concentrator pressure (250–300 kPa) and patient delivery system (regulator, filter, rotameters). By connecting the reservoir to the high-pressure side of the concentrator, the LPR prototype maintained the concentrator user interface and required no additional actions from health care workers at the time of a power outage. We think that health care workers may save valuable time and disturbances to patient care may be minimized with this approach compared to conventional high-pressure cylinder backups. The size of the reservoir likely means that it will need to be located outside the patient care area. Several oxygen storage systems exist with their own benefits and limitations. Diamedica (UK) utilizes a compressor system and a separate tank with rotameter. This unit has the advantage of storing oxygen at a higher pressure (~500 kPa) using a smaller storage volume that is transportable. Rassool [39] developed a low pressure (~16 kPa) bladder system that utilizes the pressure generated from the concentrator outlet to lift water from a lower bladder to a second bladder placed at some height above. The water applies a pressure to oxygen captured in the lower bladder to drive flow during a power outage. The LPR system leverages storage pressure in between these two approaches, enabling a design that can deliver flow from a rigid tank without additional compression or the need for a ballast system. There is a single hose connection from the concentrator to the reservoir, making it relatively easy to setup.

Additional work will be needed to build on the technical assessment presented here. Clinical studies are needed to quantify the health impact of such a technology. Additional engineering and a market assessment will also be needed to design according medical device standards and inform the value proposition. Design for manufacturing could drive down costs, as the materials used in this study were chosen for flexibility and convenience and not to minimize cost. Incorporating this system into health centers will also require integration with appropriate agencies and in concert with recommended practices and equipment to treat patients. Proper use and maintenance of the concentrator remains an important component to manage; we suspect that additional maintenance of the LPR components will include maintaining cleanliness, but this will require further evaluation. The need and cost to properly secure the vessel should also be considered.

## Conclusions

The results of this study demonstrate the ability of a novel reservoir system to store and deliver oxygen from a concentrator during a power outage and, consequently, minimize health worker intervention typically required to switch to a back-up cylinder source. The low-pressure system was able to deliver oxygen in 56% of power outage minutes and cover over 99% of power outage events. These results demonstrate an alternative, relatively simple, low cost and accessible method to extend oxygen availability. Future work will aim to explore this system in a clinical study and to assess its health and economic impact.

## Supporting information

S1 Fig Minutes of data recorded per day. (TIF)

## References

[1] GBD 2015 Mortality and Causes of Death Collaborators. Global, regional, and national life expectancy, all-cause mortality, and cause-specific mortality for 249 causes of death, 1980–2015: a systematic analysis for the Global Burden of Disease Study 2015. Lancet. 2016;388: 1459–1544. 10.1016/S0140-6736(16)31012-1 27733281 PMC5388903

[2] Beeson HD, Smith SR, Stewart WF. Safe Use of Oxygen and Oxygen Systems: Handbook for Design, Operation, and Second Edition. 2009.

[3] Junge S, Palmer A, Greenwood BM, Kim Mulholland E, Weber MW. The spectrum of hypoxaemia in children admitted to hospital in The Gambia, West Africa. Trop Med Int Heal. 2006;11: 367–372. 10.1111/j.1365-3156.2006.01570.x 16553917

[4] Subhi R, Adamson M, Campbell H, Weber M, Smith K, Duke T, et al The prevalence of hypoxaemia among ill children in developing countries: a systematic review. Lancet Infect Dis. 2009;9: 219–227. 10.1016/S1473-3099(09)70071-4 19324294

[5] Duke T, Mgone J, Frank D. Hypoxaemia in children with severe pneumonia in Papua New Guinea. Int J Tuberc Lung Dis. 2001;5: 511–519. 11409576

[6] Duke T, Blaschke AJ, Sialis S, Bonkowsky JL. Hypoxaemia in acute respiratory and non-respiratory illnesses in neonates and children in a developing country. Arch Dis Child. 2002;86: 108–112. 10.1136/adc.86.2.108 11827904 PMC1761078

[7] Mwaniki MK, Nokes DJ, Ignas J, Munywoki P, Ngama M, Newton CRJC, et al Emergency triage assessment for hypoxaemia in neonates and young children in a Kenyan hospital: An observational study. Bull World Health Organ. 2009;87: 263–270. 10.2471/BLT.07.049148 19551234 PMC2672576

[8] World health organization. Oxygen therapy for children [Internet]. 2016. Available: http://apps.who.int/iris/bitstream/10665/204584/1/9789241549554_eng.pdf?ua=1

[9] Walters EM, Benson JD, Woods EJ, Critser JK. The history of sperm cryopreservation. 2009;

[10] Graham H, Tosif S, Gray A, Qazi S, Campbell H, Peel D, et al Providing oxygen to children in hospitals: a realist review. 2017; 288–302. 10.2471/BLT.16.186676 PMC5407252 28479624

[11] Nabwire J, Namasopo S, Hawkes M. Oxygen availability and nursing capacity for oxygen therapy in Ugandan paediatric wards. J Trop Pediatr. 2017;0: 1–7. 10.1093/tropej/fmx033 28486654

[12] Enarson P, Vincente L, Gie R, Chokani C. Implementation of an oxygen concentrator system in district hospital paediatric wards throughout Malawi. Bull World Heal Organ. 2008;86: 344–348. 10.2471/BLT.07.048017 PMC2647442 18545736

[13] Ginsburg AS, Cleve WC Van, Thompson MIW, English M. Oxygen and Pulse Oximetry in Childhood Pneumonia- A Survey of Healthcare Providers in Resource-limited Settings. J Trop Pediatr. 2012;58: 389–393. 10.1093/tropej/fmr103 Advance 22170511

[14] Hill SE, Njie O, Sanneh M, Jallow M, Peel D, Njie M, et al Oxygen for treatment of severe pneumonia in The Gambia, West Africa: a situational analysis. Int J Tuberc Lung Dis. 2009;13: 587–593. 19383191

[15] Belle J, Cohen H, Shindo N, Lim M, Velazquez-Berumen A, Ndihokubwayo JB, et al Influenza preparedness in low-resource settings: A look at oxygen delivery in 12 african countries. J Infect Dev Ctries. 2010;4: 419–424. 10.3855/jidc.859 20818088

[16] Towey R, Ojara S. Practice of intensive care in rural Africa: an assessment of data from northern Uganda. Afri Heal Sci. 2008;8: 61–64. PMC2408542 19357737

[17] World Health Organization. Technical specification for oxygen concentrators. Geneva; 2015.

[18] Howie SRC, Hill S, Ebonyi A, Krishnan G, Njie O, Sanneh M, et al Meeting oxygen needs in Africa: An options analysis from the Gambia. Bull World Health Organ. 2009;87: 763–771. 10.2471/BLT.08.058370 19876543 PMC2755310

[19] Dobson MB. Oxygen concentrators offer cost savings for developing countries- a study based on Papua New Guinea. Anaesthesia. 1991;46: 217–219. 2014901 10.1111/j.1365-2044.1991.tb09413.x

[20] Mokuolu OA, Ajayi OA. Use of an oxygen concentrator in a Nigerian neonatal unit: economic implications and reliability. Ann Trop Paediatr. 2002;22: 209–212. 10.1179/027249302125001499 12369483

[21] Perrelet A, Zellweger J, Talla I, Ndiaye Y, Gautier E, Gehri M. The oxygen concentrator: an appropriate technology for treating hypoxaemic children in developing countries. Int J Tuberc Lung Dis. 2004;8: 1138–1141. 15455602

[22] Bradley BD, Light JD, Ebonyi AO, N’Jai PC, Ideh RC, Ebruke BE, et al Implementation and 8-year follow-up of an uninterrupted oxygen supply system in a hospital in The Gambia. Int J Tuberc Lung Dis. 2016;20: 1130–1134. 10.5588/ijtld.15.0889 27393551 PMC4937752

[23] Shrestha BM, Singh BB, Gautam MP, Chand MB. The oxygen concentrator is a suitable alternative to oxygen cylinders in Nepal. Can J Anest. 2002;49: 8–12. 10.1007/BF03020412 11782322

[24] Dobson MB. Oxygen concentrators and cylinders. Int J Tuberc Lung Dis. 2001;5: 520–523. 11409577

[25] Dobson M, Peel D, Khallaf N. Field trial of oxygen concentrators in upper Egypt. Lancet. 1996;347: 1597–1599. 8667871 10.1016/s0140-6736(96)91080-6

[26] Peel D, Neighbour R, Eltringham RJ. Evaluation of oxygen concentrators for use in countries with limited resources. Anaesthesia. 2013;68: 706–712. 10.1111/anae.12260 23654218

[27] Bradley BD, Chow S, Nyassi E, Cheng YL, Peel D, Howie SRC. A retrospective analysis of oxygen concentrator maintenance needs and costs in a low-resource setting: experience from The Gambia. Health Technol (Berl). 2015;4: 319–328. 10.1007/s12553-015-0094-2

[28] Matai S, Peel D, Wandi F, Jonathan M, Subhi R, Matai S, et al Implementing an oxygen programme in hospitals in Papua New Guinea. Ann Trop Paediatr. 2008;28: 71–78. 10.1179/146532808X270716 18318953

[29] Duke T, Peel D, Graham S, Howie S, Enarson PM, Jacobson R, et al Oxygen concentrators: a practical guide for clinicians and technicians in developing countries. Ann Trop Paediatr. 2010;30: 87–101. 10.1179/146532810X12637745452356 20522295

[30] Catto AG, Zgaga L, Theodoratou E, Huda T, Nair H, Arifeen S El, et al An evaluation of oxygen systems for treatment of childhood pneumonia. BMC Public Health. BioMed Central Ltd; 2011;11: S28 10.1186/1471-2458-11-S3-S28 21501446 PMC3231901

[31] Adair-Rohani H, Zukor K, Bonjour S, Wilburn S, Kuesel AC, Hebert R, et al Limited electricity access in health facilities of sub-Saharan Africa: a systematic review of data on electricity access, sources, and reliability. Glob Heal Sci Pract. 2013;1: 249–261. 10.9745/GHSP-D-13-00037 PMC4168575 25276537

[32] Schneider G. Oxygen supply in rural africa: a personal experience. Int J Tuberc Lung Dis. 2001;5: 524–526. 11409578

[33] Turnbull H, Conroy A, Opoka RO, Namasopo S, Kain KC, Hawkes M. Solar-powered oxygen delivery: proof of concept. Int J Tuberc Lung Dis. 2016;20: 696–703. 10.5588/ijtld.15.0796 27084827

[34] Nyende S, Conroy A, Opoka RO, Namasopo S, Kain KC, Mpimbaza A. Solar-powered oxygen delivery: study protocol for a randomized controlled trial. Trials. Trials; 2015;16: 297 10.1186/s13063-015-0814-y 26156116 PMC4496955

[35] Duke T, Hwaihwanje I, Kaupa M, Karubi J, Panauwe D, Sa M, et al Solar powered oxygen systems in remote health centers in Papua New Guinea: a large scale implementation effectiveness trial. J Glob Health. 2017;7: 010411 10.7189/jogh.07.010411 28567280 PMC5441450

[36] Graham HR, Ayede AI, Bakare AA, Oyewole OB, Peel D, Gray A, et al Improving oxygen therapy for children and neonates in secondary hospitals in Nigeria: Study protocol for a stepped-wedge cluster randomised trial. Trials. Trials; 2017;18: 1–12. 10.1186/s13063-016-1752-z 29078810 PMC5659007

[37] Bradley BD, Qu S, Cheng Y-L, Peel D, Howie SRC. Options for Medical Oxygen Technology Systems in Low-Resource Settings: A Framework for Comparison. 2012 IEEE Glob Humanit Technol Conf. 2012; 356–362. 10.1109/GHTC.2012.53

[38] Duke T, Peel D, Wandi F, Subhi R, Sa’avu M, Matai S. Oxygen supplies for hospitals in Papua New Guinea: a comparison of the feasibility and cost-effectiveness of methods for different settings. P N G Med J. 2010;53: 126–138. 23163183

[39] Rassool RP, Sobott BA, Peake DJ, Mutetire BS, Moschovis PP, Black JF. A Low-Pressure Oxygen Storage System for Oxygen Supply in Low-Resource Settings. Respir Care. 2017; respcare.05532. 10.4187/respcare.05532 28951467 PMC6373847

[40] ASTM. Standard Guide for Designing Systems for Oxygen Service. Annu B ASTM Stand. 2005;14.04,: 418–422. 10.1520/g0088

[41] ISO 15001 Anaesthetic and respiratory equipment—Compatibility with oxygen. 2010.

[42] Farooq S, Ruthven DM, Boniface HA. Numerical simulation of a pressure swing adsorption oxygen unit. Chem Eng Sci. Pergamon; 1989;44: 2809–2816. 10.1016/0009-2509(89)85090-0

