# Supplementary figures and images for: Assessment of a storage system to deliver uninterrupted therapeutic oxygen during power outages in resource-limited settings
Source: PLoS One. 2019 Feb 6;14(2):e0211027. doi: 10.1371/journal.pone.0211027 (PMC6364892; doi:10.1371/journal.pone.0211027)

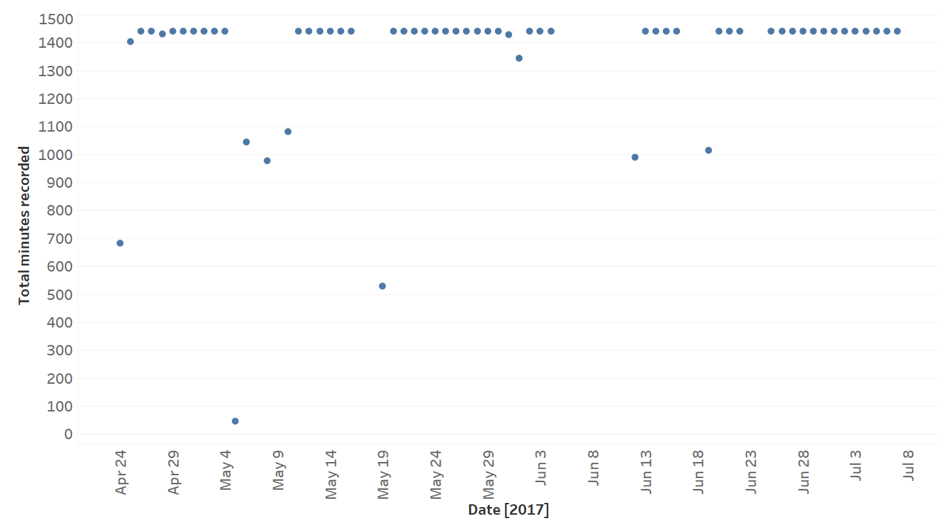

Supplement: S1 Fig — (TIF) [file pone.0211027.s001.tif]
